# Species composition, infection rate and detection of resistant alleles in *Anopheles funestus* (Diptera: Culicidae) from Lare, a malaria hotspot district of Ethiopia

**DOI:** 10.1186/s12936-023-04667-3

**Published:** 2023-08-12

**Authors:** Delelegn Woyessa, Evangelia Morou, Nadja Wipf, Nsa Dada, Konstantinos Mavridis, John Vontas, Delenasaw Yewhalaw

**Affiliations:** 1 Department of Biology, College of Natural Sciences, Jimma University, P. O. Box 378, Jimma, Ethiopia; 2 Institute of Molecular Biology and Biotechnology, Foundation for Research and Technology-Hellas, 70013 Heraklion, Greece; 3 Swiss Tropical and Public Health Institute, Allschwil, Switzerland; 4 University of Basel, Basel, Switzerland; 5 School of Life Sciences, College of Liberal Arts and Sciences, Arizona State University, Tempe, USA; 6 School of Medical Laboratory Sciences, Faculty of Health Sciences, Jimma University, Jimma, Ethiopia; 7 Tropical and Infectious Diseases Research Centre (TIDRC), P.O. Box 378, Jimma, Ethiopia

**Keywords:** *Anophelus funestus*, Resistant allele, Genotyping, Secondary malaria vectors, Single nucleotide polymorphisms, Ethiopia

## Abstract

**Background:**

*Anopheles funestus,* which is considered as secondary vector of malaria in Ethiopia, is known to have several morphologically indistinguishable (sibling) species. Accurate identification of sibling species is crucial to understand their biology, behaviour and vector competence. In this study, molecular identification was conducted on the Ethiopian *An. funestus* populations. Moreover, insecticide resistance mechanism markers were detected, including *ace* N485I, *kdr* L1014F, L1014S, and CYP6P9a TaqMan qPCR was used to detect the infective stage of the parasite from field collected adult female *An. funestus* populations.

**Methods:**

Adult female mosquito collection was conducted from Lare, Gambella Regional State of Ethiopia between June 2018 to July 2020 using CDC light traps and HLC. Sub-samples of the morphologically identified *An*. *funestus* mosquitoes were molecularly identified using species-specific PCR, and the possible presence of insecticide resistance alleles was investigated using TaqMan qPCR (N485I-Ace-1), PCR-Sanger sequencing (L1014F-kdr), and PCR–RFLP (CYP6P9a resistance allele). Following head/thorax dissection, the TaqMan qPCR assay was used to investigate the presence of the infective stage *Plasmodium* parasite species.

**Results:**

A total of 1086 adult female *An*. *funestus* mosquitoes were collected during the study period. All sub-samples (N = 20) that were morphologically identified as *An. funestus *sensu lato (*s.l*.) were identified as *An. funestus *sensu stricto (*s.s*.) using species- specific PCR assay. The PCR–RFLP assay that detects the CYP6P9a resistance allele that confers pyrethroid resistance in *An. funestus* was applied in N = 30 randomly selected *An. funestus s.l.* specimens. None of the specimens showed a digestion pattern consistent with the presence of the CYP6P9a resistance allele in contrast to what was observed in the positive control. Consequently, all samples were characterized as wild type. The qPCR TaqMan assay that detects the N485I acetylcholinesterase-1 mutation conferring resistance to organophosphates/carbamates in *An. funestus* was used in (N = 144) samples. All samples were characterized as wild type. The *kdr* L1014F and L1014S mutations in the VGSC gene that confer resistance to pyrethroids and DDT were analysed with direct Sanger sequencing after PCR and clean-up of the PCR products were also characterized as wild type. None of the samples (N = 169) were found positive for *Plasmodium (P*. *falciparum*/*ovale*/*malariae*/*vivax)* detection.

**Conclusion:**

All *An. funestus s.l.* samples from Lare were molecularly identified as *An. funestus s.s*. No CYP6P9, N485I acetylcholinesterase 1, *kdr* L1014F or L1014S mutations were detected in the *An. funestus* samples. None of the *An. funestus* samples were positive for *Plasmodium*. Although the current study did not detect any insecticide resistant mechanism, it provides a reference for future vector monitoring programmes. Regular monitoring of resistance mechanisms covering wider geographical areas of Ethiopia where this vector is distributed is important for improving the efficacy of vector control programs.

## Background

Malaria continues to be a burden in the sub-Saharan African region [1] regardless of significant case declines in the past decades as a result of interventions, mainly using indoor-based long-lasting insecticidal nets (LLINs) and indoor residual spraying (IRS) [2–5]. In the World Health Organization (WHO) African region, an estimated 215 million malaria cases and 384,000 deaths in 2021 account for 94% of the global burden [6]. Malaria is the leading cause of mortality and morbidity in Ethiopia and about 75% of the total area of the country is malarious and an estimated 68% of the people are at risk of malaria [7].

In Africa, particularly in sub-Saharan Africa, of 140 anopheline species, only about 20 of them are known to transmit malaria to humans [8–10]. According to previous study [11], five species, namely, *Anopheles gambiae*, *Anopheles arabiensis*, *Anopheles funestus*, *Anopheles moucheti* and *Anopheles nili*, are considered to be major malaria vectors which are responsible for 95% of the total malaria transmission on the continent while the remaining (5%) is transmitted by “secondary” or “vectors of local importance”.

Though secondary vectors of malaria are usually considered to play minor role in malaria transmission and have limited distribution, they may contribute significant role in local transmission as they potentially extend malaria transmission period [12–15]. Accordingly, historical research focus and transmission characterization on ‘primary’ vectors may result in a biased dataset especially in the context of intervention-based impacts on susceptible primary vectors [1]. In addition, the continued use of indoor interventions and consequent selective pressures on primary vectors may have differential impacts on secondary vectors that have different bionomics [1].

In Ethiopia, target site resistance mechanism has been reported in populations of *An. arabiensis*, the principal malaria vector and the *kdr* allele frequency of the L1014F mutation in the Gilgel Gibe region was the highest ever reported from this vector species [16]. *Anopheles funestus* is also considered as major malaria vector in sub-Saharan Africa [11, 17], but, historically, it is a secondary malaria vector in Ethiopia [18]. *Anopheles funestus* is known to have several morphologically indistinguishable (sibling) member species. Such morphologically indistinguishable cryptic species are challenges to malaria control as their biology and role in malaria transmission is less understood [19]. Previously, *An. funestus* group has been reported from Gojjam, northwestern Ethiopia [20] and Bure District [18]. However, information about species composition and the distribution of the different *An. funestus* group in the country has not yet been reported. There is also paucity of data on insecticide resistance status and infection rates in *An. funestus* group in the country. Therefore, accurate identification of sibling species is crucial to understand their biology, ecology, behaviour and hence vector competence [21]. Speciation of *An. funestus* group*,* characterization of insecticide resistance mechanisms and detection of pathogens in *An. funestus* are important for designing appropriate future vector control strategy. Hence, this study aimed at identifying member species of *An. funestus* using molecular assays. Moreover, characterizing of insecticide resistance mechanisms and detecting of pathogen in populations of *An. funestus* malaria vector in Ethiopia.

## Methods

### Study area

**Fig. 1:**
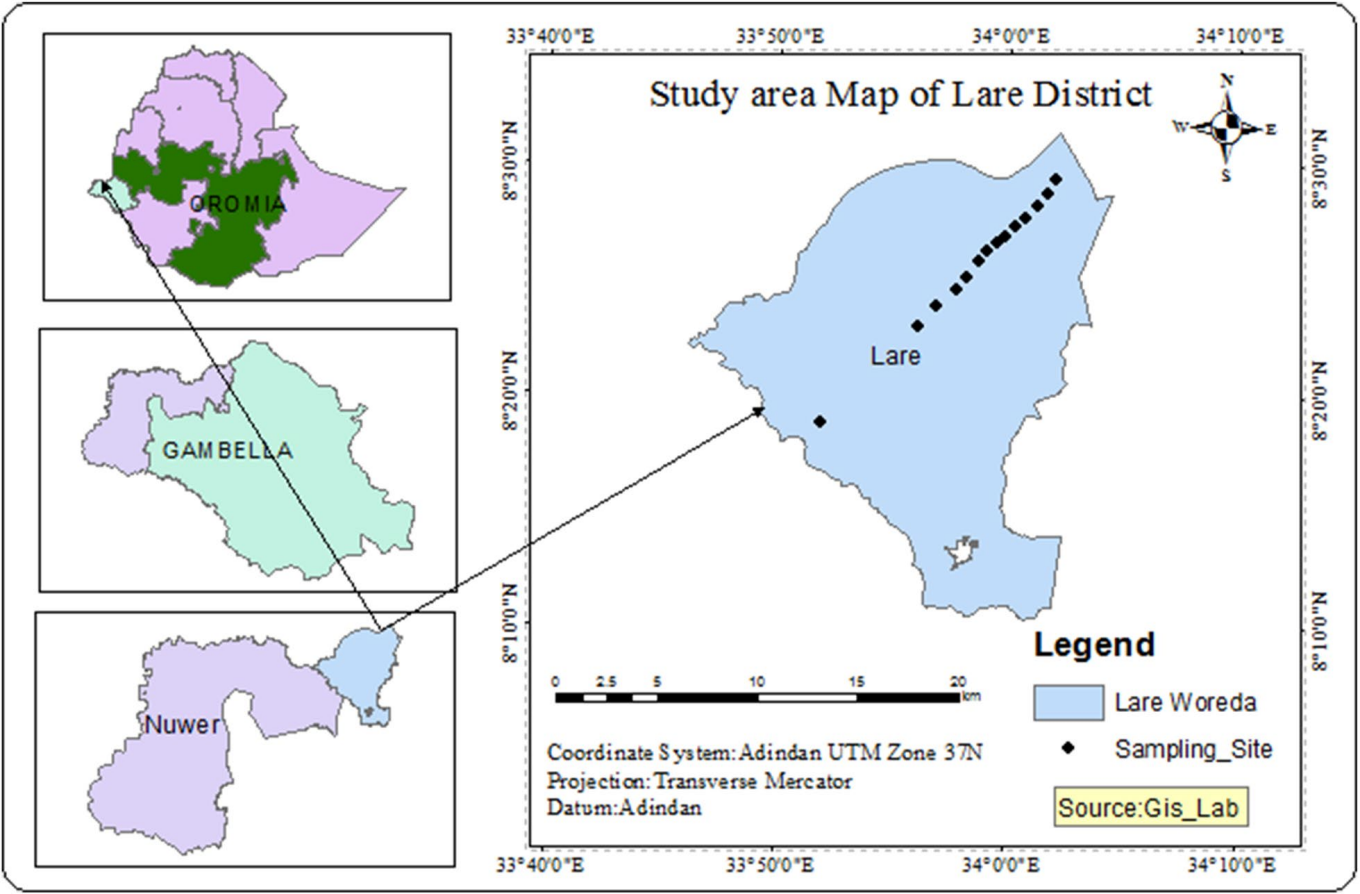
Map of the study area

A longitudinal study design was employed to collect adult mosquitoes from Lare district (Woreda), Gambella Regional State, Ethiopia (Fig. 1). Lare is one of the Districts in Nuer Zone, in the Gambela Regional State of Ethiopia. Lare is located in south-western tip of Ethiopia at about 780 km from the capital, Addis Ababa and 89 km from Gambella, the Regional State Capital. It is bordered on the west by the Baro River, which separates it from Jikawo, on the north by the Jikawo River which separates it from South Sudan and on the south and east by Anuak Zone. The landscape consists of marshes and tall grasses. The district receives an average rainfall of 1,900–2,100 mm. The temperature rises, up to 45 °C during summer in March; and in August, during the rainy season of June to November, it reaches 27–31 °C. Part of Lare is also located within Gambella National Park, which occupies part of the area south of the Baro River. The main livelihood of the population is pastoralism and mixed farming. The people are largely dependent on livestock. They also raise goats and rarely sheep.

### Ethical clearance

An institutional ethical approval with Ref. No. IHRPGD/787/2019 was obtained from Institutional Review Board (IRB) of Institute of Health, Jimma University. Informed written consent was obtained from trained volunteers who participated in mosquito collections. Head of families were requested through individual discussions and group meetings, prior to the selection of their house for mosquito collection in the study. Permission was requested and obtained from inhabitants to conduct mosquito collections both indoor and outdoor.

### Mosquito collection

Adult female Anopheles mosquito collection was conducted in Lare, Ethiopia from June 2018 to July 2020 using CDC light traps (BioQuip Model 2836BQ, USA) and human landing catches (HLC). Sample collection was conducted using CDC light traps (CDC LTs) in every month for three consecutive nights for a total of 12 months between June 2018 and July 2020 from four randomly selected mud constructed animal sheds. The CDC miniature light traps were suspended approximately about 1.5 m above the floor. Traps were switched at 18:00 PM and switched off at 6:00 AM. Traps were collected from each house every morning at 6:00 AM and mosquitoes were killed using cotton wool treated with chloroform. Mosquitoes were also collected using human landing couches (HLC) in another four randomly selected mud constructed houses. The HLC was conducted overnight with simultaneous indoor and outdoor collections. The HLC operation was performed for three consecutive nights of collection in each house during every month, yielding a total of 24 person-nights (12 indoor and 12 outdoor) each month.. There were two teams of 4 trained people consented for mosquito collection. Four collectors performed the collection indoor and four outdoor. The HLC was performed between 6:00 PM and 6:00 AM. The period of collection was divided into 6-h segments; thus, two of the four trained volunteers for indoor for 6-h collection as well as outdoor collectors were worked for 6 h (6:00 PM to mid-night) and sleep 6 h alternatively while two of the remaining perform collections from mid-night to 6:00 AM. The team conducted the collection every night. The collectors wore long-sleeve shirts during the collections to prevent mosquitoes landing and biting on arms [22].

For molecular profiling of the field collected adult female *An. funestus,* the following experimental plan was used: Adult female *An. funestus s*.*l*. were collected from Lare District of Gambella Regional State. The molecular experiments conducted included PCR-agarose gel electrophoresis for species identification, TaqMan qPCR for Ace N485I and *Plasmodium* detection, PCR-sanger sequencing for Kdr L1014F/S and PCR–RFLP for CYP6P9a R allele assays. *Anopheles funestus* FANG (Insecticide susceptible laboratory strain) and Fumoz (insecticide resistant laboratory strain) were used as controls for detection of insecticide resistance mechanism study.

### Genomic DNA extraction from mosquitoes

Samples of adult *An. funestus* mosquitoes (N = 313 in total) collected using HLC and CDC light traps from Lare, Ethiopia, were used to extract genomic DNA using the DNazol protocol, according to the manufacturer’s instructions (Molecular Research Center Inc). The quantity and purity of DNA was assessed using spectrophotometry via Nano drop measurements.

### Molecular identification of An. funestus

The Wilkins et al. [23] MR4 PCR assay was used for molecular identification of the *An. funestus* samples in a subsample of N = 20 randomly selected *An. funestus* mosquitoes. The MR4 method [24] of *An. funestus* complex discrimination is based on species-specific single nucleotide polymorphisms (SNPs) in the second internal transcribed spacer region (ITS2). Primers that create fragments of 496 bp *Anopheles vaneedeni*, 424 bp *An. funestus s.s*., 346 bp, *Anopheles rivulorum*, 241 bp *Anopheles rivulorum*-like (West Africa), 176 bp *Anopheles parensis* and 93 bp *Anopheles leesoni* were used*.* The detailed protocol and primers are described in Table 1.

**Table 1:** Primers probes and protocols used for the assay

Assay	Primers/Probes 5'–3'	Reaction protocol	Thermal cycle	PCR product
A. *funestus* Species ID	FUNUNIV_F: CCGATGCACACATTCTTGAGTGCCTA FUN _R: CTCGGGCATCGATGGGTTAATCATG VAN _R: AACTCTGTCGACTTGGTAGCCGAAC RIV_R: AATCAGGGTCGAACGGCTTGCCG PAR_R: GCCCTGCGGTCCCAAGCTAGATT RIVLIKE_R: CTCCCGTGGAGTGGGGGATC LEES_R: GACGGCATCATGGCGAGCAGC	KapaTaq [500 nM primers] 2.0 mM MgCl2	94 °C/4 min × 1 cycle (94 °C/30 s 58 °C/30 s, 72 °C/45 s) × 30 cycles; 72 °C/7 min × 1 cycle	496 bp *An. vaneedeni*, 424 bp *An. funestus*, 346 bp, *An. rivulorum*, 241 bp *An. rivulorum*-like (West Africa), 176 bp *An. parensis* and 93 bp *An. leesoni* [run 10 μl sample]
Kdr detection	F: GGMGAATGGATYGAATCMATGTGGGA R: GATGAACCRAAATTKGACAAAAGCAA 3'	KapaTaq [500 nM primers]	94 °C/ 5 min, 94 °C/ 1 min, 50 °C/ 2 min, 72 °C/ 2 min (35 cycles), 72 °C/ 2 min	200 bp
CYP6P9a	F: 5′-TCCCGAAATACAGCCTTTCAG-3′, R: 5′-ATTGGTGCCATCGCTAGAAG	PCR: KapaTaq; 30 uL reaction [500 nM primers]	The 30 μl solution underwent a denaturing step at 95 °C for 5 min, followed by 35 cycles of 94 °C for 30 s, 57 °C for 30 s and 72 °C for 1 min and 30 s, followed by a final extension step of 72 °C for 10 min	450 bp [run 10 uL, keep the rest for digestion]
		Digestion: 20 uL final volume [10.0 uL PCR product; 1 uL from 10U TaqI; 2 uL TaqI buffer 10x; BSA up to 100 ug/mL; water up to 20 uL]	Digestion: Incubate at 65 °C for 2 h	Restriction digest was separated on 2.0% agarose gel. The Taq I enzyme cut the 450-bp fragment from the putative pyrethroid resistance haplotype into two fragments of 350 and100 bp
N485I acetylcholinesterase1 detection	F: CATGCGATACTGGTCAAACTTTGC R: GCCATTCGGGAAATTCGCTACTA P (wt) HEX-CAAACCCCAACACGGC-MGB P (mut): FAM-CAAACCCCATCACGGC-MGB	The TaqMan reactions were performed in a 10 uL final volume containing 1 × TaqManunivesalmastermix (Applied Biosystems), 800 nM of each primer and 200 nM of each probe,	Cycling conditions were: 10 min at 95 °C, 40 cycles of 15 s at 92 °C and 1 min at 60 °C	N/A
*Plasmodium* detection	F: GCTTAGTTACGATTAATAGGAGTAGCTTG R: GAAAATCTAAGAATTTCACCTCTGACA P: Falcip + (FAM-TCTGAATACGAATGTC-MGB) P: OVM + (HEX-CTGAATACAAATGCC-MGB')	The TaqMan reactions were performed in a 10 uL final volume containing 1 × TaqManunivesalmastermix (Applied Biosystems), 800 nM of each primer and 300 nM of each probe	Cycling conditions were: 10 min at 95 °C, 45 cycles of 15 s at 92 °C and 1 min at 60 °C	N/A

### Assessment of resistance markers (CYP6P9a allele, N485I acetylcholine esterase 1 and kdr L1014F/S)

The detailed protocols and primers used for detection of target site mutations are described in Table 1. Accordingly, PCR–RFLP assay [25] was used for the detection of the CYP6P9a resistant allele in N = 30 randomly selected samples. Restriction site (5′-TCGA-3′) for the Taq I enzyme at the A/G mutation located 18 bp of the AA insertion and completely tight with the CCAAT box on the resistance haplotype was used to design a PCR–RFLP assay to genotype the CYP6P9aR allele. The TaqMan assay previously developed [26] was used for the detection of N485I acetylcholine esterase 1 mutation in N = 144 randomly selected samples. The presence of *kdr* L1014F/S was investigated using the PCR assay (KAPA Taq PCR Kit; KAPA Biosystems, Wilmington, MA, US) described in Table 1 in N = 20 randomly selected samples. PCR amplicons were purified or extracted from agarose gels using the Nucleospin PCR & Gel Clean-Up Kit (Macherey Nagel) and sequenced with the Sanger method (CeMIA S.A., Larissa, Greece).

### Assessment of the infective stage of Plasmodium

The heads and thoraces of mosquitoes to be analysed for *P. falciparum*/*ovale*/*malariae*/*vivax* were dissected in order to detect the presence of the infective (sporozoite) stage of the *Plasmodium* parasite species. A total of 169 *An. funestus* specimens, selected based on sample availability, were assayed with TaqMan qPCR as per an established protocol [27] used in Table 1.

### Data analysis

For mosquitoes collected using HLC, the man-biting rate (ma) was expressed as the number of bites a person receives from a specific vector species per night [28]. Accordingly, human biting rate (HBR) = number of mosquitoes collected ÷ number of collectors ÷ number of nights. Since four houses were selected for HLC sampling and four collectors were involved per house for three consecutive nights, the man-biting rate was calculated as number of *An. funestus* collected divided by four collectors divided by three nights. Density/house-night was determined thus by dividing total female *An. funestus* to sampled number of houses per number of consecutive nights in each collection month. Sanger sequencing results for *kdr* detection were analysed using the sequence alignment editor BioEdit 7.2.5. Analysis of resistance markers status CYP6P9a allele [25], N485I acetylcholine esterase 1 [26] and Plasmodium detection [27] was performed as described in the established protocols.

## Results

### Anopheles funestus: density, human biting rate and molecular species identification

**Fig. 2:**
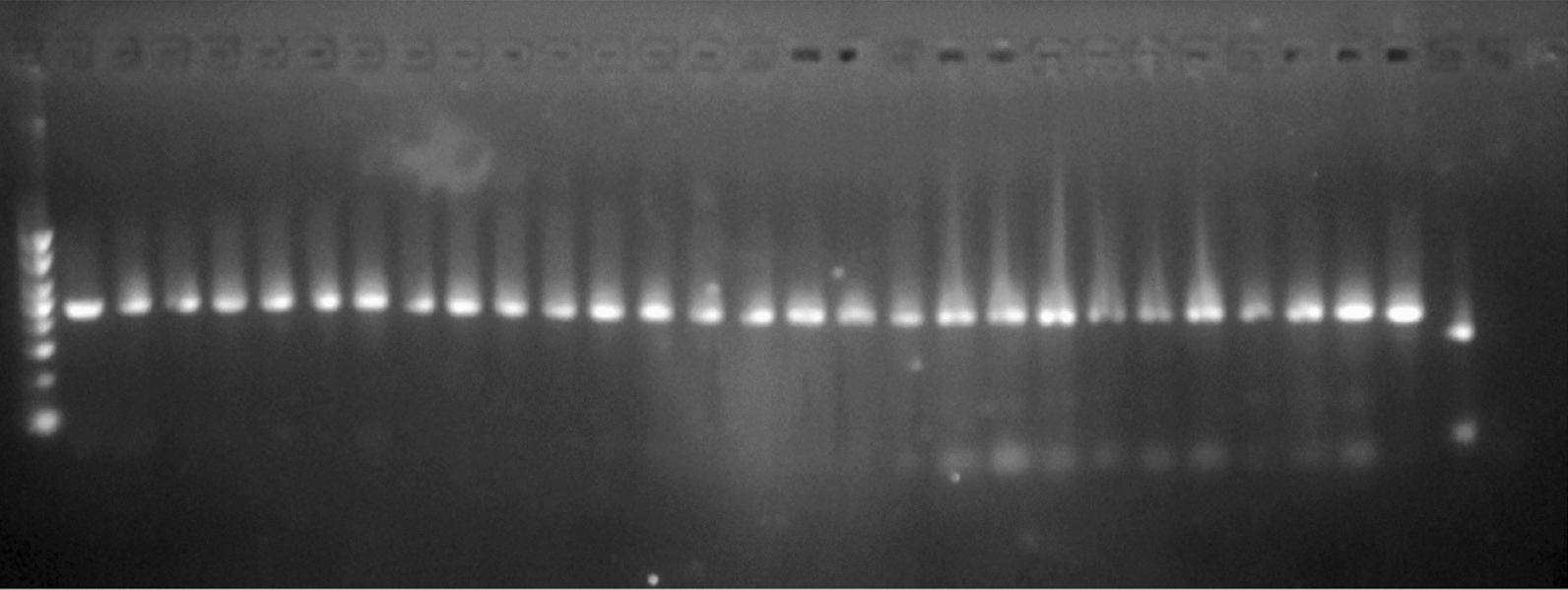
Gel electrophoresis of *An. funestus* identification of following established [24] protocol (1st Lane: Molecular Weight DNA marker, 2nd Lane: Positive Control, last lane: Negative control)

A total of 1086 Adult female *An. funestus* mosquitoes were collected during the study period. Of which 63% (N = 684) were captured by HLC while the rest, 37% (N = 402) were captured by CDC light traps (Table 2). A total of 20 (1.8%) sub-samples of *An. funestus s.l.* were molecularly identified and all belonged to *An. funestus s.s.* (Fig. 2).

**Table 2:** Density and human biting rate of *Anopheles funestus* s*.l.* collected from Lare District, Gambella, Ethiopia (June 2018-July 2020)

Collection Month-Year	Method of collection & number of captured *Anopheles funestus s.l*		Total captured	Density/house-night	Human-biting rate (HBR)
	CDC	HLC			
Jun. 2018	5	0	5	0.42	0.00
Sept. 2018	0	2	2	0.17	0.17
Oct. 2018	0	34	34	2.83	2.83
Nov. 2018	0	3	3	0.25	0.25
Dec. 2018	288	523	811	67.58	43.58
Jan. 2019	45	122	167	13.92	10.17
Feb. 2019	0	0	0	0.00	0.00
Mar. 2019	1	0	1	0.08	0.00
Aug. 2019	0	0	0	0.00	0.00
Sept. 2019	0	0	0	0.00	0.00
Nov. 2019	63	0	63	5.25	0.00
Jul.2020	0	0	0	0.00	0.00
Total	402	684

### Monitoring for CYP6P9a and acetylcholine esterase mutations

**Fig. 3:**
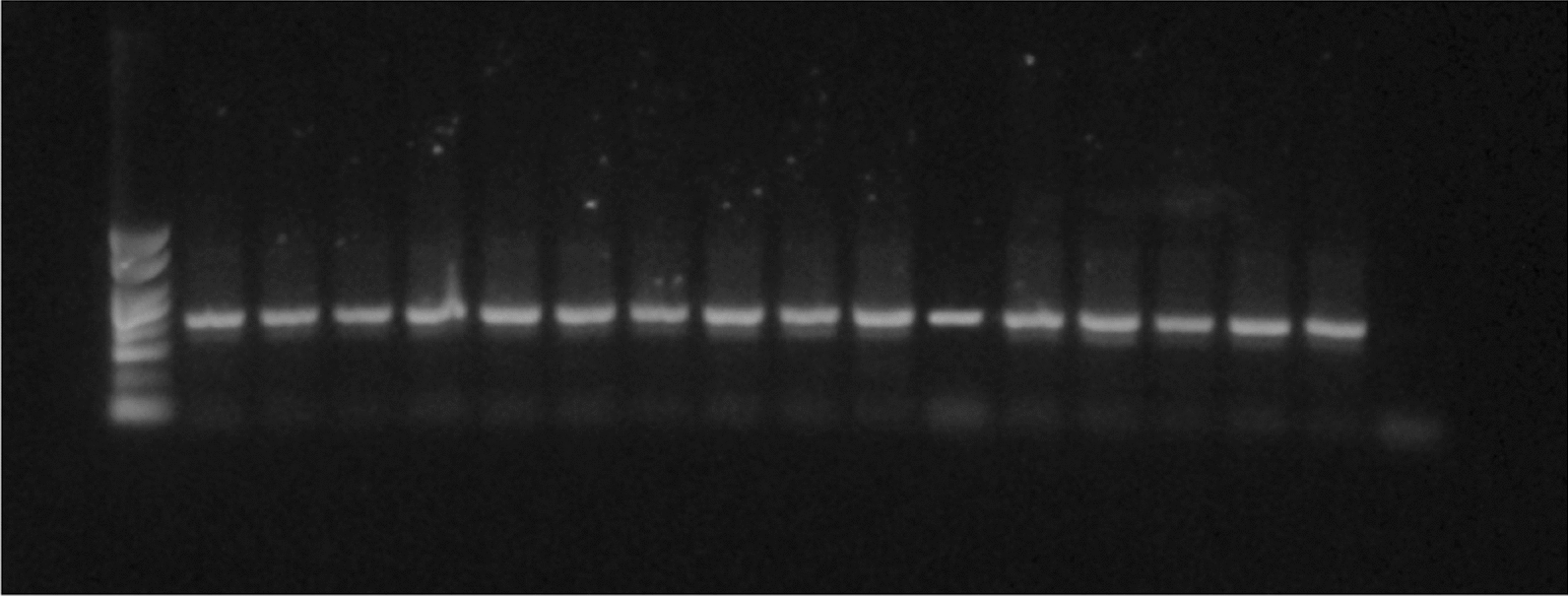
Results of gel electrophoresis of *An. funestus* samples characterized for the presence of the CYP6P9a resistance allele (1st Lane: Molecular Weight DNA marker, last lane: Positive control-Fumoz resistant lab strain)

The PCR–RFLP assay that detects the CYP6P9a resistant allele which confers pyrethroid resistance in *An. funestus* mosquitoes was applied in N = 30 randomly selected *An. funestus* specimens collected from Lare, Ethiopia. None of the specimens showed a digestion pattern consistent with the presence of the CYP6P9a resistant allele (Fig. 3), which is observed in the positive control (last lane). Consequently, all samples were characterized as wild type.

**Fig. 4:**
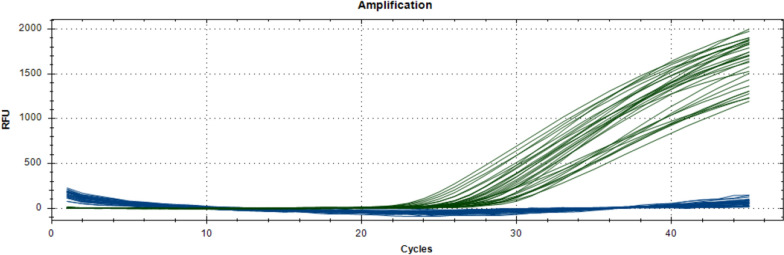
Reaction curves of *An. funestus* assayed for the detection of N485I acetylcholinesterase-1 mutation

The qPCR TaqMan assay employed to detect the N485I acetylcholinesterase-1 mutation conferring resistance to organophosphates/carbamates in *An. funestus* was used in N = 144 samples. And all samples were characterized as wild type (Fig. 4).

**Fig. 5:**
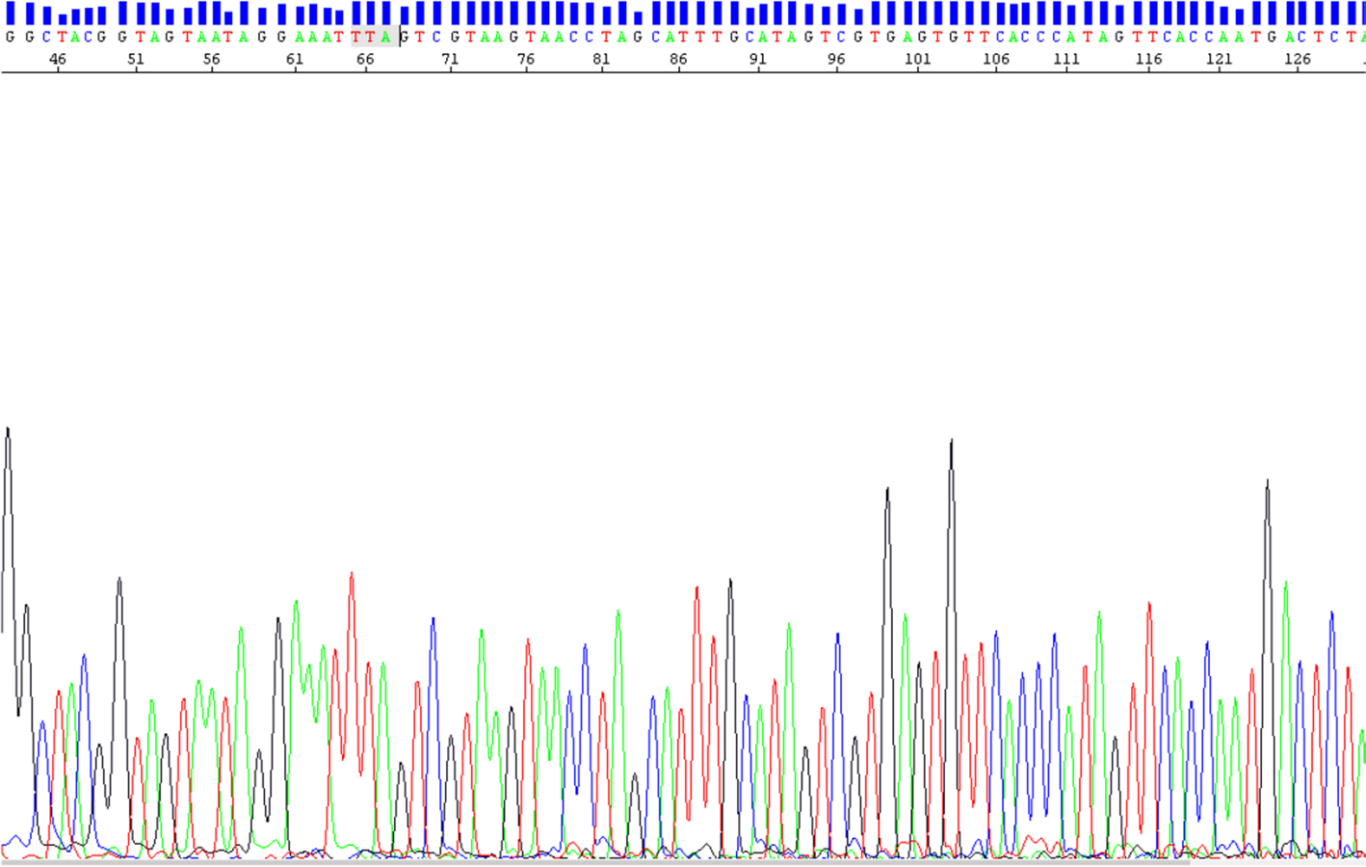
Electropherogram of DNA sequencing of VGSC gene fragment of *An. funestus* sample used for the possible detection of *kdr* L1014F/S mutation

The *kdr* L1014F and L1014S mutations in the VGSC gene that confer resistance to pyrethroids and DDT were analysed with direct Sanger sequencing after PCR and clean-up of the PCR products. All *An. funestus* samples (N = 20) were characterized as wild type (TTA-L) (Fig. 5).

### Monitoring for Plasmodium infection

All dissected heads and thoraces of *An. funestus* mosquitoes (N = 169) that were processed were found negative for the presence of the infective (sporozoite) stage of *P. falciparum*/*ovale*/*malariae*/*vivax* parasites.

## Discussion

In this study, the Ethiopian *Anopheles funestus* s.l. population was molecularly identified to the species level. A subset of samples randomly selected from the morphologically identified *An. funestus s.l*. mosquitoes revealed the exclusive presence of *An. funestus s.s*. Although the sub-samples of mosquitoes assayed to identify sibling species of *An. funestus* group was low, the sub-samples were randomly selected from all *An. funestus* samples collected in different seasons of the study period. Hence, the fact that all the samples tested were identified as *An. funestus s.s.* could suggest that *An. funestus s.s.* may possibly be the predominant sibling species of *An. funestus* group occurring in the area. Moreover, detection of sporozoite infection and characterization of insecticide resistance mechanisms were performed on samples from Lare, south-western Ethiopia.

Although *An. funestus* is considered as the primary malaria vectors in other sub-Saharan African countries [29], however, it is a secondary malaria vector in Ethiopia [18]. Collections using both CDC and HLC confirmed higher abundance of this vector species during the dry season (December-January), which was following the peak malaria season. Hence, the vector could be responsible to extend the malaria transmission period and potentially sustain malaria transmission after the density of the principal vectors declined by vector control interventions, such as IRS or LLINs. This could be one of the major challenges to malaria elimination programme as December-January period was when most (90%) *An. funestus* were captured and highest density/house/night was recorded. The period was post IRS use against the principal vectors whose density peaks in October and November [30] and hence there was no insecticide spray in the study area. Other studies [31, 32] also reported that *An. funestus* follows the peak abundance season of its counterpart, *An. gambiae s.l*, therefore, extending malaria transmission from the beginning to the first part of the dry season [31, 32]. Similar to the current finding, Charlwood et al. [33] have also reported that *An. funestus* is the most important dry season malaria vector in Savannah part of East Africa.

In the current study, CYP6P9a resistance allele, N485I acetylcholinesterase 1, *kdr* L1014F or L1014S mutations were not detected in *An. funestus*. Hence, molecular analysis of sub-samples from the collected mosquitoes to detect resistant alleles and subsequent absence of resistance alleles following analysis could indicate the absence of such mutations in mosquito populations in the study area. Contrary to the current study, multiple insecticide resistance mechanisms in *An. funestus* were previously reported in different localities in Benin, West Africa [34, 35]; East Africa [36]. Furthermore, Riveron et al. [37] demonstrated that DDT detoxification in *An. funestus* is mainly associated with a single mutation in the GSTe2 gene. Resistance to at least four classes of insecticides by *An. funestus* populations have also been reported from many African countries [38–41]. In the current study, we did not investigate L119F-GSTe2 mutation, which could probably contribute for resistance as reported elsewhere from Kenya and Uganda [36], and it would be important to include in future monitoring studies to better understand the resistance mechanisms in *An. funestus* populations of Ethiopia.

None of the *An. funestus* samples were found positive for *Plasmodium* detection in the current study. This could be explained by the change in biting behaviour as a result of being confronted by insecticide-based interventions in the study area, although this finding could be impacted by the relatively small number of mosquito samples examined.

## Conclusion

Collections using both CDC and HLC confirmed higher abundance of *An. funestus* mosquitoes following the peak malaria season (December-January) from Lare District of Ethiopia. This may sustain malaria transmission after the density of the principal vectors declined by vector control interventions, such as IRS or LLINs. Molecular identification of randomly selected *An. funestus s.l*. samples collected in different seasons from Lare resulted in the identification of *An. funestus s.s*. Although the current study did not detect insecticide resistant mechanism, possibly due to the small sample size available for analysis, it provides a reference for future vector monitoring programs. However, absence of insecticide resistant alleles in the current *An. funestus* samples may not necessarily represent the whole picture of Ethiopian *An. funestus* populations. Hence, regular monitoring of resistance mechanisms that will involve wider geographical areas of Ethiopia where this vector is distributed is important for improving the efficacy of vector control programmes.

## Data Availability

The data used are available from the corresponding author up on request.
